# NORMSEQ: a tool for evaluation, selection and visualization of RNA-Seq normalization methods

**DOI:** 10.1093/nar/gkad429

**Published:** 2023-05-22

**Authors:** Chantal Scheepbouwer, Michael Hackenberg, Monique A J van Eijndhoven, Alan Gerber, Michiel Pegtel, Cristina Gómez-Martín

**Affiliations:** Department of Neurosurgery, Cancer Center Amsterdam, Amsterdam University Medical Center (UMC) location Vrije Universiteit Amsterdam, Amsterdam 1081HV, The Netherlands; Cancer Center Amsterdam, Cancer Biology, Amsterdam, The Netherlands; Department of Pathology, Cancer Center Amsterdam, Amsterdam UMC location Vrije Universiteit Amsterdam, Amsterdam 1081HV, The Netherlands; Genetics Genetics Department, Faculty of Science, Universidad de Granada, Campus de Fuentenueva s/n, 18071, Granada, Spain; Bioinformatics Laboratory, Biomedical Research Centre (CIBM), Biotechnology Institute, PTS, Avda. del Conocimiento s/n, 18100 Granada, Spain; Excellence Research Unit “Modeling Nature” (MNat), University of Granada, Spain; Instituto de Investigación Biosanitaria ibs.GRANADA, University Hospitals of Granada-University of Granada, Spain, Conocimiento s/n, 18100, Granada, Spain; Department of Pathology, Cancer Center Amsterdam, Amsterdam UMC location Vrije Universiteit Amsterdam, Amsterdam 1081HV, The Netherlands; Cancer Center Amsterdam, Imaging and Biomarkers, Amsterdam, The Netherlands; Department of Neurosurgery, Cancer Center Amsterdam, Amsterdam University Medical Center (UMC) location Vrije Universiteit Amsterdam, Amsterdam 1081HV, The Netherlands; Cancer Center Amsterdam, Cancer Biology, Amsterdam, The Netherlands; Department of Pathology, Cancer Center Amsterdam, Amsterdam UMC location Vrije Universiteit Amsterdam, Amsterdam 1081HV, The Netherlands; Cancer Center Amsterdam, Imaging and Biomarkers, Amsterdam, The Netherlands; Department of Pathology, Cancer Center Amsterdam, Amsterdam UMC location Vrije Universiteit Amsterdam, Amsterdam 1081HV, The Netherlands; Cancer Center Amsterdam, Imaging and Biomarkers, Amsterdam, The Netherlands

## Abstract

RNA-sequencing has become one of the most used high-throughput approaches to gain knowledge about the expression of all different RNA subpopulations. However, technical artifacts, either introduced during library preparation and/or data analysis, can influence the detected RNA expression levels. A critical step, especially in large and low input datasets or studies, is data normalization, which aims at eliminating the variability in data that is not related to biology. Many normalization methods have been developed, each of them relying on different assumptions, making the selection of the appropriate normalization strategy key to preserve biological information. To address this, we developed NormSeq, a free web-server tool to systematically assess the performance of normalization methods in a given dataset. A key feature of NormSeq is the implementation of information gain to guide the selection of the best normalization method, which is crucial to eliminate or at least reduce non-biological variability. Altogether, NormSeq provides an easy-to-use platform to explore different aspects of gene expression data with a special focus on data normalization to help researchers, even without bioinformatics expertise, to obtain reliable biological inference from their data. NormSeq is freely available at: https://arn.ugr.es/normSeq.

## Graphical Abstract

**Graphical Abstract:**
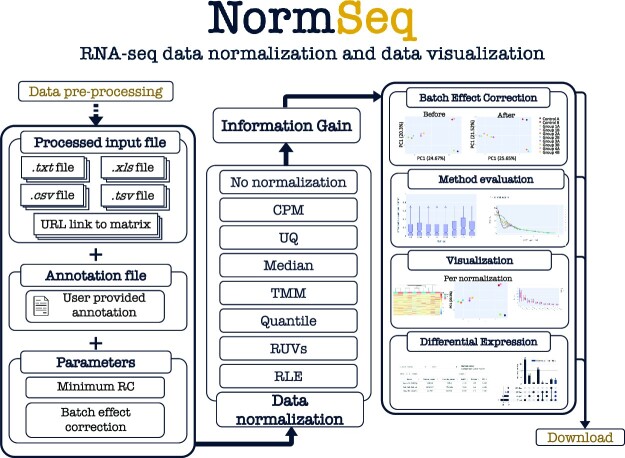

## INTRODUCTION

The continuous improvement of RNA sequencing (RNA-seq) methodologies (1–3), together with the reduction in sequencing costs, has resulted in a significant rise in both the number of RNA-seq studies as well as the size of biological datasets. This opened up new possibilities for RNA discovery and profiling, along with in-depth studies of genes behaviour under different biological and pathological conditions (4).

Although high-throughput RNA sequencing offers valuable insights into disease biology, it can also be subjected to various non-biological technical biases, which can result in differences in sequencing depth (5,6), or GC-content (7), among others. Consequently, an essential step during RNA-seq data analysis is the selection of an appropriate normalization strategy to remove unwanted variation caused by technical artefacts. The application of the correct normalization method is crucial to recover biological signal, i.e. the truly differentially expressed genes, while avoiding incorrect biological inference further downstream of the analysis.

Initially, intra-sample ‘normalization by library size’ methods (8,9), like Reads per Kilobase per Million (RPKM), Fragments per Kilobase per Million (FPKM) or Counts per Million (CPM), were frequently applied, while nowadays, cross-sample distribution based methods such as Trimmed mean of M values (TMM), quantile normalization (QN), Relative log expression (RLE/DEseq) or Median Ratio Normalization (MRN) are usually employed. This latter group of methods seeks to determine a scaling factor that is applied to the raw read counts, correcting for sequencing depth and stabilising variation between samples (10). Yet, another set of methods relies on the existence of control genes (5) (spike-ins, housekeeping genes) and were initially developed to correct for batch effects, i.e. variation that is introduced when samples are processed and sequenced in separate batches.

It is important to realise that all methods rely on certain assumptions that need to be met, otherwise the number of false positive and negative differentially expressed (DE) genes will increase. Key assumptions for distribution-based methods are that only a few DE genes exist and technical artefacts affect DE and non-DE genes in the same way (11). Over the last years considerable efforts have been made to compare the performance of the different normalization methods (11–13) and to select the most appropriate normalization for each situation (14,15). Strong differences do exist in the performance of the methods depending on the experimental design and the studied biological conditions (see Table 2 from Evans *et al.* (11)), which determine whether the underlying assumptions are met or not. However, in practice it is not easy to infer the best normalization strategy directly from a given experimental design. Different tools exist that combine several normalization methods and downstream analysis, such as GENAVi (16). However, to our best knowledge, there is no user-friendly tool available dedicated to the assessment of normalization methods for RNA-seq datasets and the evaluation of its impact on downstream analysis.

To this end, we developed NormSeq, a freely accessible webserver tool that is dedicated to the evaluation and direct comparison of the most commonly used data normalization methods for any user-supplied RNA-seq expression dataset. The goal of NormSeq is to systematically compare normalization approaches and guide the user towards the best normalization method, i.e. the one that correctly recovers biological signal. We implemented the use of the information gain metric to guide the selection of the most appropriate normalization method and RLE plots for a visual inspection of the normalization results. Moreover, NormSeq includes the possibility of performing batch-effect correction, as well as multiple downstream analyses, such as consensus differential gene expression, multiple visualizations and the download of all the information available on the webserver.

NormSeq is available at: https://arn.ugr.es/normSeq.

## WORKFLOW AND SCOPE

**Figure 1.**
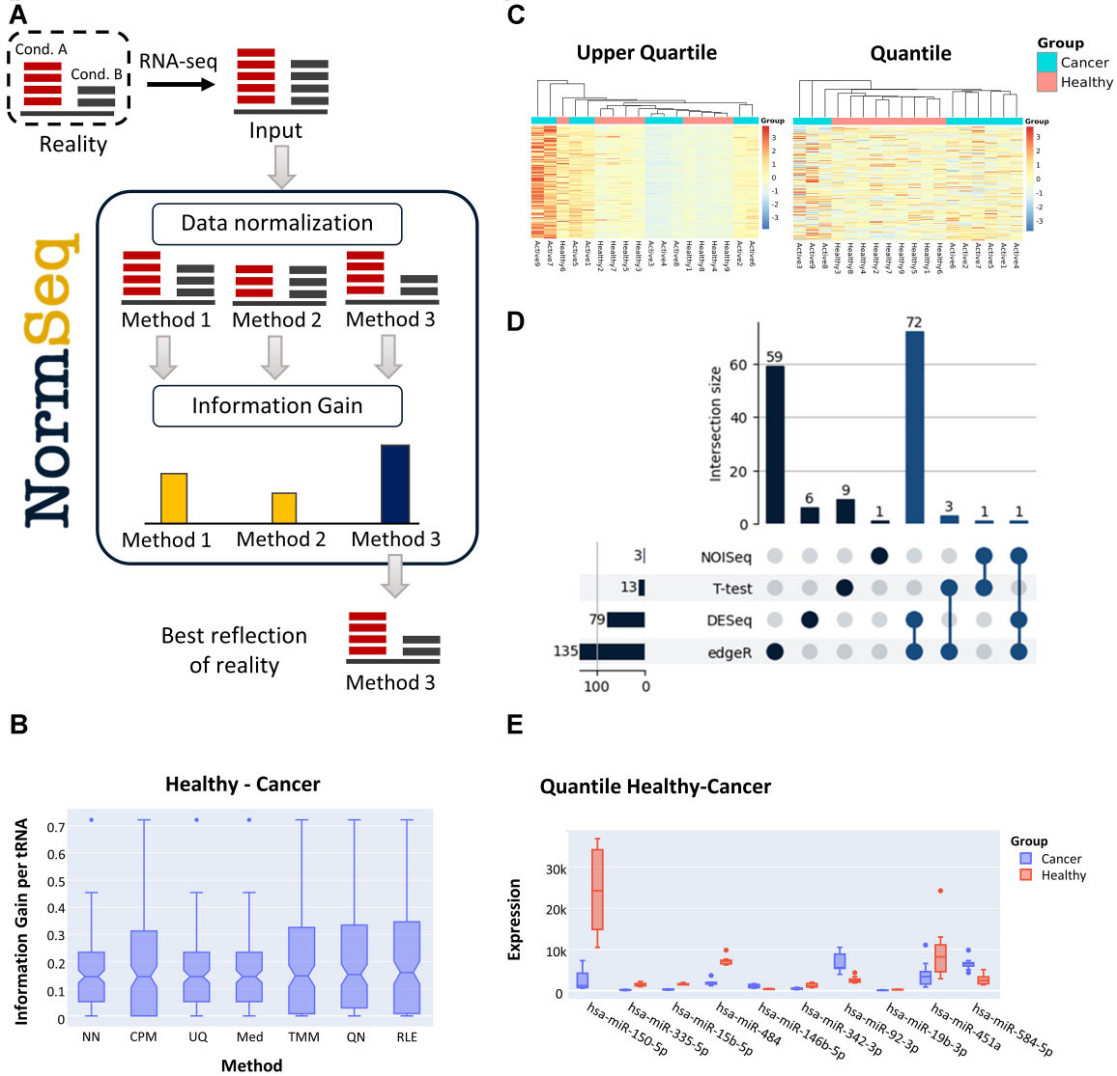
NormSeq's workflow and implementation. (**A**) Workflow of NormSeq. User-provided RNA-seq counts are used for data normalization. NormSeq provides eight different options for data normalization, four differential expression analysis protocols, and optional batch effect correction. Assessment based on the information gain distribution guides selection of the best normalization method that helps obtain the most reliable biological inference from the data. (**B**) Information gain distribution of seven out of eight of the normalization methods available in NormSeq applied to the miRNA sequencing dataset SRP326090 (32). The comparison of healthy individuals and cancer patients with active Hodgkin Lymphoma is shown, where 4 methods (CPM, TMM, QN and RLE) outperformed the others in terms of information gain. (**C**) Hierarchical clustering analysis of the miRNA seq data in healthy individuals and cancer patients with active disease. Data is normalized by upper quartile (left) and quantile (right), showing that quantile normalization clusters better represent the two biological conditions. (**D**) Upset plot showing the intersection of differentially expressed miRNAs detected with edgeR, DESeq2, NOISeq and a Student's *t*-test. (**E**) Boxplot visualization of the top 10 highest fold change miRNAs between healthy individuals and cancer patients.

Different experimental conditions and designs call for different normalization methods (11). NormSeq's main aim is to provide researchers with an easy-accessible and systematic approach towards RNA-seq data normalization. To this end, a side-by-side evaluation is implemented for the most commonly used normalization methods: Counts Per Million (CPM), Upper Quantile (UQ), Median (Med), Trimmed mean of M values (TMM), Quantile (QN), Relative Log Expression (RLE) and Remove Unwanted Variation in its RUVs version (see Table 1 for more details). One of NormSeq's main innovations is the implementation of the information gain distribution analysis, in order to select the best normalization method for each given dataset. Additionally, to visually inspect the outcome of the different normalization methods, we have included RLE plots, that can give hints on the amount of unwanted variation removed with each of them. The general workflow of NormSeq (Figure 1A) includes the normalization of the user-provided RNA-seq counts, information gain per RNA distribution assessment and finally a large subset of downstream analysis, such as clustering analysis, PCA and DE-analysis.

**Table 1.** Description of the normalization methods implemented in NormSeq

Normalization method	Description	Reference
Counts per million (CPM)	CPM normalization corrects for library size without considering transcript length. Each read count is divided by the total read count, followed by multiplying by 1 000 000	Dillies, Brief Bioinfor, 2013 (9)
Upper quartile (UQ)	All genes with a read count of 0 are removed, followed by a division of the remaining gene counts by the upper quartile	Bullard, Bioinfor, 2010 (8)
Median (Med)	Median normalization adjusts the data of each individual sample by adding a constant value to achieve the same median value across all samples	Dillies, Brief Bioinfor, 2013 (9)
Trimmed mean of M values (TMM)	The TMM method estimates scale factors for comparing libraries on a relative scale	Robinson, Genome Biol, 2010 (6)
Quantile (QN)	Quantile normalization applies a mathematical transformation to the rank statistics across samples	Bolstad *et al.*, Bioinfor, 2003 (10)
Remove unwanted variation (RUVs)	RUVs estimates the factors of unwanted variation using replicate samples	Risso *et al.*, Nat Biotech, 2014 (5)
Relative log expression (RLE)	For each gene, the RLE scaling factor is computed as the median of the ratio of the read counts by taking the geometric mean across all samples	Anders, Genome Biol, 2010 (22)

## NORMSEQ INPUT AND TOOLS OVERVIEW

### Data input and normalization method selection

The normalization analysis starts with user-provided datasets that are required to be presented as raw count tables. Users can supply the count matrix by directly uploading a file in *txt*, *csv*, *tsv* or *xls* formats or by providing a URL link to the file in one of those formats. A matched annotation file is mandatory to initiate the normalization analysis on the webserver, and users have the possibility to provide an additional batch-effect annotation file. Several parameters can be adjusted to personalize the normalization analysis. This includes the selection of a minimum read coverage, the choice for computational batch effect correction, and differential expression analysis related parameters. A separate job will be created for each normalization analysis with a URL that will remain active for 15 days.

### Information gain, RNA expression distributions and RLE plots

A unique feature of NormSeq is the possibility to select the most optimal normalization method for a given dataset by means of the information gain (also called mutual information), an information theory method (17) (Figure 1B). In essence, the information gain quantifies the degree of mutual dependence of two continuous variables by the reduction in impurity or randomness for each RNA based on its expression levels across all samples, while considering the biological groups to which they belong. Information gain has been described as a well-suited statistical metric for this purpose based on several qualities. First, it is capable to detect any kind of relationship between datasets, regardless if it involves the mean values, the variance or higher moments. Secondly, it has a straightforward interpretation, as it is expressed as a value between 0 (highest impurity, lowest information gain) and 1 (lowest impurity, highest information gain), where higher values of information gain would represent a clear dependence between the levels of expression of a given RNA and the biological groups. And finally, it is insensitive to the dataset size, which differs from other statistical tests that are depending on the size of datasets to a much larger extent for the evaluation of statistical significance, even for poorly related variables.

The challenge in computing information gain in RNA-seq datasets resides mainly in the fact that the underlying probability distribution of the data is not known, and the continuous normalized data needs to be transformed from a continuous scale to a discrete probability distribution. Different methods exists to do this transformation. Among them, we selected the ‘Nearest Neighbour’ transformation that has been described as optimal for this type of experimental design (17,18).

NormSeq offers the information gain distribution for all chosen normalization methods in two formats: for each pair-wise group comparison and for each individual group. For pair-wise group comparisons (Figure 1B), a normalization method with a higher information gain would better recover the biological signal for that particular comparison. On the other hand, when considering information gain distribution per group, a higher information gain distribution would indicate that the differences of that group compared to all other groups are more apparent using that specific method.

**Figure 2.**
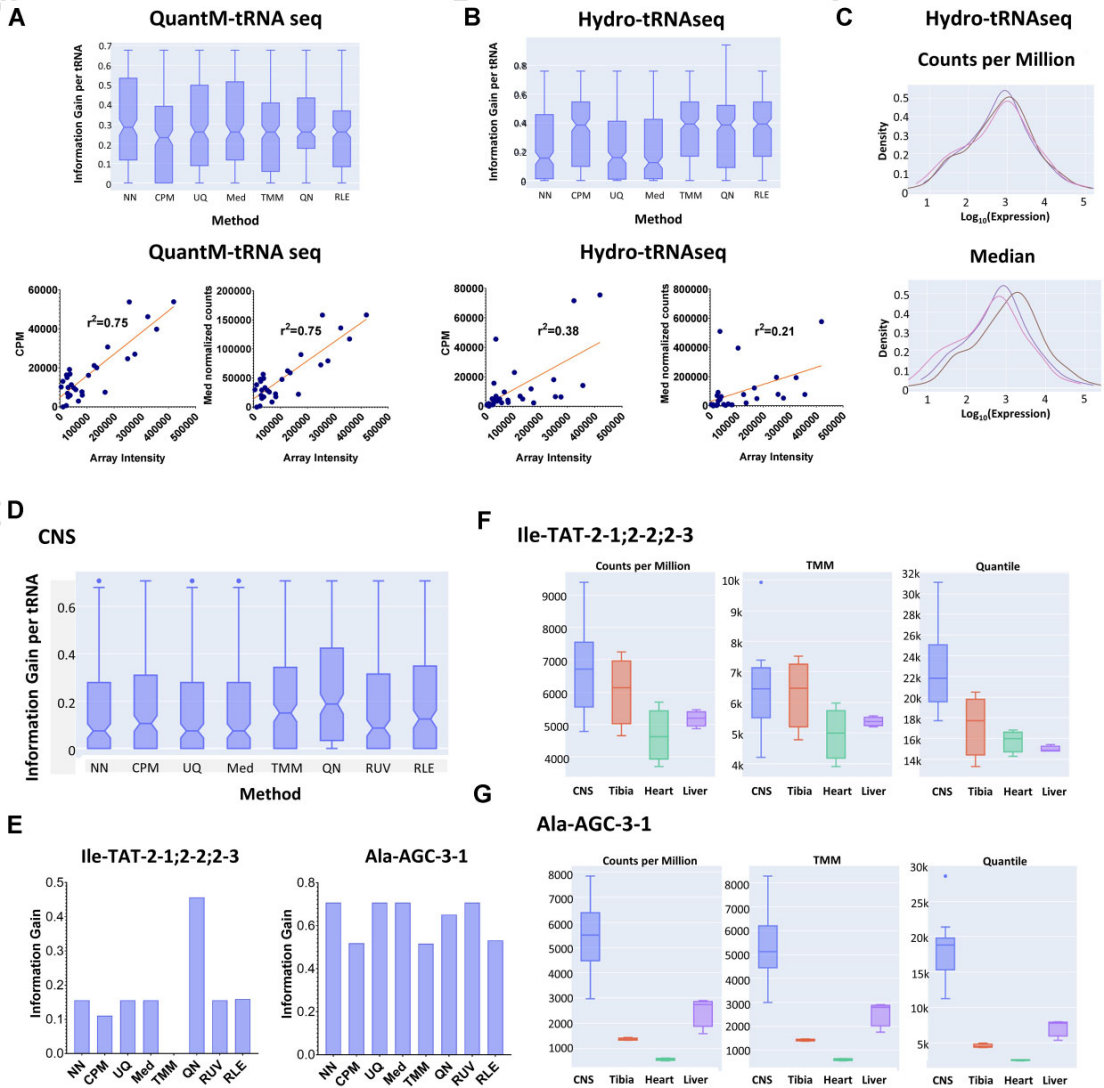
NormSeq can guide users in selecting the most appropriate normalization method for every dataset. (**A**) (top panel) Notched boxplot of information gain results for NormSeq's normalization methods (no normalization (NN), counts per million (CPM), upper quartile (UQ), median (Med), trimmed mean of *M* values (TMM), quartile (QN) and relative log expression (RLE)) applied to count tables from QuantM-tRNA seq data in HEK293T cells. (bottom panel) Pearson correlation of CPM and Med normalized read counts QuantM-tRNA seq data versus tRNA array quantification. (**B**) (top panel) Notched boxplot of information gain results for NormSeq's normalization methods (no normalization, CPM, UQ, Med, TMM, QN and RLE) applied to count tables from Hydro-tRNAseq data in HEK293T cells. (bottom panel) Pearson correlation of CPM and median normalized Hydro-tRNAseq data versus tRNA array quantification. (**C**) RNA expression distribution for CPM (top panel) and Med (bottom panel) normalization. Data are represented as log_10_ values on the x-axis. (**D**) Notched boxplot of information gain results for NormSeq's normalization methods (no normalization, CPM, UQ, Med, TMM, QN, RUVs and RLE) applied to count tables from QuantM-tRNA seq data in CNS tissues. (**E**) Bar graph showing the information gain for brain-enriched tRNA genes tRNA-Ile-TAT-2–1;2–2;2–3 (left panel) and tRNA-Ala-AGC-3–1 (right panel). (**F**) Box plot showing the comparison of CPM, TMM, and QN normalization for tRNA-Ile-TAT-2–1;2–2;2–3 expression in CNS, tibia, heart and liver tissues from the QuantM-tRNA seq dataset. (**G**) Box plot showing the comparison of CPM, TMM and QN normalization for tRNA-Ala-AGC-3–1 expression in CNS, tibia, heart, and liver tissues from the QuantM-tRNA seq dataset.

Besides the information gain analysis, the RNA expression distribution is shown (Figure 2C). This is a reflection of the expression levels of the different RNAs in each sample. Distributions between groups need to be comparable in order to increase the probability of correct biological inferences for further downstream analysis. Therefore, normalization methods that lead to very dissimilar distributions for the different samples would potentially not be suitable as the detected differences could be due to technical biases.

Finally, NormSeq offers RLE plot visualization, which is included to assess and estimate the unwanted variation removal of each method. All together, these tools assist users in the selection of the most optimal normalization method for downstream analysis of their particular dataset.

### Visualization: heatmap, PCA, top expressed RNAs and per feature plots

NormSeq offers a multitude of visualization options that can be personalized and downloaded. The visualization section of the NormSeq webserver is divided into three sections. Hierarchical clustering analyses are incorporated for side-by-side visualization of similarities between samples for each chosen normalization method (Figure 1C). Principal component analysis (PCA) is implemented for the exploration of each normalization method chosen in a low-dimensional state. Furthermore, comprehensive downstream analysis of RNA expression is facilitated by plots showing the individual RNA expression levels per normalization method, as well as plots representing the top 10 most expressed RNAs, and the RNAs with the highest fold changes (FC) per comparison.

### Batch effect correction

Batch effect correction using the ComBat-Seq tool (19) is offered in the NormSeq environment. Users can upload a matrix containing the potential batch effects for correction of the data. The differences of clustering metrics in the dataset before and after the batch effect correction are visualized in a PCA plot. Subsequently, the batch-effect corrected matrix is used for the normalization assessment.

### Differential expression analysis

Differential expression (DE) analysis is one of the most commonly used applications of RNA-seq data analysis (20). NormSeq implements 4 methods to detect differentially expressed RNAs: edgeR (21), DESeq2 (22,23), NOISeq (24) and a Student's *t*-test. We provide a summary with under-, and over-expressed RNAs for each DE protocol. The consensus differential expression between all methods is calculated and can be visualized with Upset plots (Figure 1D). Additionally, results for each DE method can be accessed individually or in summary, and a visualization of the top 10 differentially expressed genes is included (Figure 1E).

## WORKING EXAMPLE

To illustrate the usefulness of our normalization tool, we analyzed a publicly available tRNA sequencing dataset, accession number GSE141436 from the NCBI Gene Expression Omnibus (GEO) repository (25). In this study, the authors developed a tRNA sequencing method, QuantM-tRNA seq, which was extensively validated by hybridization-based approaches, and simultaneously compared to other tRNA sequencing methods. As hybridization-based approaches are often used to confirm tRNA expression data, we first examined the correlation between the hybridization-based tRNA array quantification and the different normalizations of the tRNA sequencing data. We compared all normalization methods available in NormSeq, and we used the calculated information gain to guide the selection of normalization. Besides the CPM normalization that was used in the original study, the information gain evaluation of NormSeq's normalization approaches applied to QuantM-tRNA seq data, showed that all approaches performed similarly (Figure 2A; top panel). The similar performance of these normalization methods was also reflected by the subsequent analyses that showed a strong correlation between the normalized QuantM-tRNA seq data and the tRNA array-based quantification data for all tested normalization methods (Pearson correlation *r*: 0.75; Figure 2A, bottom panels). However, when we evaluated the information gain distribution of data from the same dataset, but obtained with an alternative protocol, hydro-tRNA seq (26), our results revealed that the information gain levels distributions were not similar for all normalizations (Figure 2B, top panel). Interestingly, this was supported by the different correlations of all considered normalization methods with the array intensities (Figure 2B; bottom panels). While CPM normalization still showed comparable correlation with the tRNA array quantification (Pearson correlation r: 0.38; Figure 2B, bottom left), the Median method performed poorly (Pearson correlation *r*: 0.21; Figure 2B, bottom right). These results were further supported by the global differences that were observed in tRNA expression distributions across the different groups in the analysis, as shown in Figure 2C, confirming that not all normalization methods are always appropriate for each study. Intrigued by these results, we aimed to further explore if the information gain could also guide the choice of normalization for the assessment of tRNA abundance with biological relevance. Therefore, we analyzed the tRNA expression profiles of 21 samples from seven different mouse tissues (central nervous system (CNS), liver, tibia and heart), from the same study. Information gain per tRNA isodecoder distribution was evaluated for each normalization method in all tissues. In this example, the quantile normalization seemed to outperform the other normalization methods according to its higher information gain distribution (Figure 2D). Whereas the tissue-restricted expression of the brain-specific tRNA-Arg-TCT-4–1 (25,27) was readily detected in the CNS samples following all normalization strategies (Supplementary Figure 1), the impact of the data chosen normalization method became apparent for the less extreme changes in tRNA expression. For this purpose, we assessed the expression of two additional, previously described, brain-enriched tRNA genes, tRNA-Ile-TAT-2–1;2–2;2–3 (28) and tRNA-Ala-AGC-3–1 (25,28). The information gain distribution of quantile (QN) normalization was the highest for tRNA-Ile-TAT-2–1;2–2;2–3, while TMM was shown to be close to 0 (Figure 2E; left panel). In contrast, tRNA-Ala-AGC-3–1 showed comparable performance for all normalizations, with a marginal increase for the UQ, Med and RUVs methods (Figure 2E; right panel). The evaluation of normalization performance using the information gain, was confirmed by the fact that TMM normalization, as well as CPM to a lesser extent, could not differentiate tRNA-Ile-TAT-2–1;2–2;2–3 expression between all tissues (Figure 2F). On the other hand, quantile normalization preserved the biological signal and successfully detected enrichment of tRNA-Ile-TAT-2–1;2–2;2–3 in the CNS samples (Figure 2F). Lastly, the brain-specific tRNA-Ala-AGC-3–1 could be readily detected by all selected methods, as predicted by the similar information gains (Figure 2G).

Taken together, the panel of tools provided by NormSeq to evaluate normalization methods clearly indicated that each normalization method perform differently for every dataset. As the performance of each normalization method heavily depends on the sequencing protocol, the design of the study, as well as the biological circumstances, the NormSeq application can guide the user in effortlessly selecting the most appropriate normalization method to better capture meaningful expression profiles of RNAs of interest.

## IMPLEMENTATION DETAILS

NormSeq website was implemented using a Django framework, together with Bootstrap and Javascript. Information gain is calculated by means of *mutual_info_classi*f function from Scikit-learn python package (29). PCA plots are computed with the Scikit-learn package with a previous scale step with the *MinMax* function from the same package. The plotly package (30), and the R package pheatmap (31) were used for data visualization in order to improve the interactivity of the web application.

## CONCLUSION AND OUTLOOK

In this manuscript we introduce NormSeq, a web server that provides users a guided normalization selection for high-dimensional and complex RNA-seq datasets. The main goal of NormSeq was to create an analytic and user-friendly platform that simplifies RNA-seq data normalization and differential expression analysis. To the best of our knowledge, NormSeq is the first web server that offers logical and stepwise navigation through seven different options for data normalization, four differential expression analysis protocols, information gain calculation and optional batch effect correction for the analysis of high-throughput RNA-seq data.

Future improvements will include incorporation of additional normalization methods and visualization options, as well as the inclusion of an optional stand-alone version of the NormSeq tool. Finally, we also plan to extend our NormSeq webserver towards the application of data generated with different high-throughput profiling platforms such as single cell RNA-sequencing.

## DATA AVAILABILITY

NormSeq is freely available for all users at: https://arn.ugr.es/normSeq/ and the source code is available at https://github.com/cris12gm/normSeq.

## Supplementary Material

gkad429_Supplemental_File
