# Supplementary material for: NORMSEQ: a tool for evaluation, selection and visualization of RNA-Seq normalization methods
Source: Nucleic Acids Res. 2023 May 22;51(W1):W372–8. doi: 10.1093/nar/gkad429 (PMC10320083; doi:10.1093/nar/gkad429)
Supplement: gkad429_Supplemental_File [file gkad429_supplemental_file.pdf]

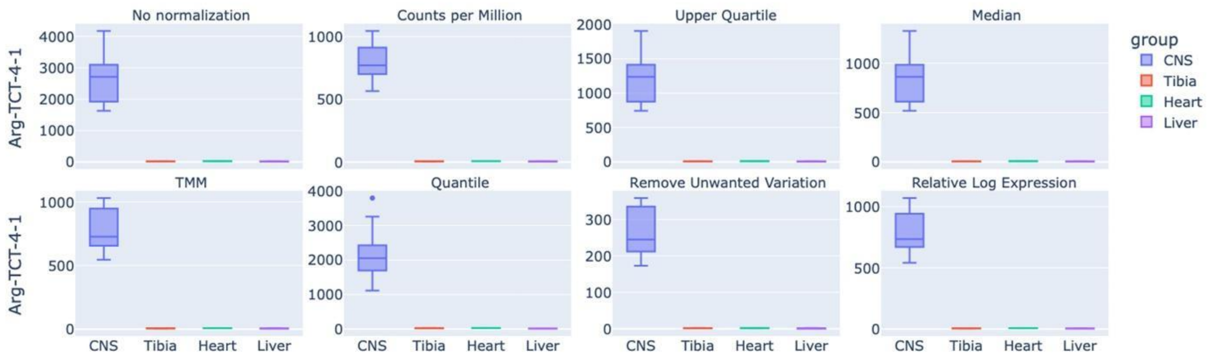

Supplementary Figure 1. Box plot showing the CNS-specific tRNA-Arg-TCT-4-1 expression in the CNS compared to tibia, heart and liver tissues from the QuantM-tRNA seq dataset with all the different normalization methods (CPM, UQ, Med, TMM, QN, RUVs and RLE).
